# Reference range for serum neurofilament light chain: findings from healthy Thai adults

**DOI:** 10.1093/braincomms/fcaf166

**Published:** 2025-04-29

**Authors:** Nontapat Sukhonpanich, Tatchaporn Ongphichetmetha, Ekdanai Uawithya, Jiraporn Jitprapaikulsan, Natthapon Rattanathamsakul, Naraporn Prayoonwiwat, Sasitorn Siritho

**Affiliations:** Division of Neurology, Department of Medicine, Faculty of Medicine Siriraj Hospital, Mahidol University, Bangkok 10700, Thailand; Division of Neurology, Department of Medicine, Faculty of Medicine Siriraj Hospital, Mahidol University, Bangkok 10700, Thailand; Siriraj Neuroimmunology Centre, Faculty of Medicine Siriraj Hospital, Mahidol University, Bangkok 10700, Thailand; Division of Clinical Epidemiology, Department of Research and Development, Faculty of Medicine Siriraj Hospital, Mahidol University, Bangkok 10700, Thailand; Siriraj Neuroimmunology Centre, Faculty of Medicine Siriraj Hospital, Mahidol University, Bangkok 10700, Thailand; Division of Neurology, Department of Medicine, Faculty of Medicine Siriraj Hospital, Mahidol University, Bangkok 10700, Thailand; Siriraj Neuroimmunology Centre, Faculty of Medicine Siriraj Hospital, Mahidol University, Bangkok 10700, Thailand; Division of Neurology, Department of Medicine, Faculty of Medicine Siriraj Hospital, Mahidol University, Bangkok 10700, Thailand; Siriraj Neuroimmunology Centre, Faculty of Medicine Siriraj Hospital, Mahidol University, Bangkok 10700, Thailand; Division of Neurology, Department of Medicine, Faculty of Medicine Siriraj Hospital, Mahidol University, Bangkok 10700, Thailand; Siriraj Neuroimmunology Centre, Faculty of Medicine Siriraj Hospital, Mahidol University, Bangkok 10700, Thailand; Division of Neurology, Department of Medicine, Faculty of Medicine Siriraj Hospital, Mahidol University, Bangkok 10700, Thailand; Siriraj Neuroimmunology Centre, Faculty of Medicine Siriraj Hospital, Mahidol University, Bangkok 10700, Thailand; Neuroscience Centre, Bumrungrad International Hospital, Bangkok 10110, Thailand

**Keywords:** neurofilament light chain, reference range, biomarker

## Abstract

Serum neurofilament light chain is a notable biomarker for detecting axonal injury and has shown significant potential for clinical applications. Establishing a reference interval and cut-off level is a critical step towards implementing a serum neurofilament light chain in routine clinical practice. In this study, we aimed to establish a reference range of serum neurofilament light chains for the Thai population. Blood samples were collected from healthy Thai adults without a history of neurological diseases and screened at the Siriraj Hospital. The relationship between age, sex and log_10_-transformed serum neurofilament light chain levels was analysed using linear regression. A crude reference interval was calculated as the 2.5–97.5th percentile values. An age-normative percentile curve for serum neurofilament light chain was derived using the generalized additive model for location, scale and shape. A total of 223 subjects (96 males and 127 females) aged 18–70 years were recruited. Male sex (*P* = 0.008) and older age (*P*  *<* 0.001) were significantly associated with higher serum neurofilament light chain levels. A median of the observed serum neurofilament light chain values was 5.8 pg/ml (95% confidence interval 5.4–6.2), ranging from 1.0 to 18.4 pg/ml, with a crude reference interval of 2.3–15.9 pg/ml. The 2.5–97.5th percentile intervals for serum neurofilament light chain by age group were as follows: 20–29 years (*n* = 57): 1.7–8.7 pg/ml; 30–39 years (*n* = 58): 2.5–10.6 pg/ml; 40–49 years (*n* = 59): 3.5–14.3 pg/ml; 50–59 years (*n* = 37): 4.7–15.8 pg/ml and 60–69 years (*n* = 12): 4.2–18.2 pg/ml. The age-normative serum neurofilament light chain curve predicted the 97.5th percentile of 8.2, 9.9, 11.7, 14.6 and 19.9 pg/ml for ages 20, 30, 40, 50 and 60, respectively. This study is the first to establish reference values for serum neurofilament light chains in Thailand. The age-normative upper reference curve is closely aligned with observed values and those previously reported in other studies, providing a robust framework for clinical implementation. However, further validation in larger cohorts and among individuals with neurological diseases is warranted.

## Teaser

This study aimed to create reference values for serum neurofilament light chain for Thai. We utilized multiple statistical methods to predict the cut-off values in healthy Thai. The age-normative cut-off curve is closely aligned with observed values and those previously reported. Further validation in patients with neurological disorders is planned.

## Graphical Abstract

**Graphical Abstract:**
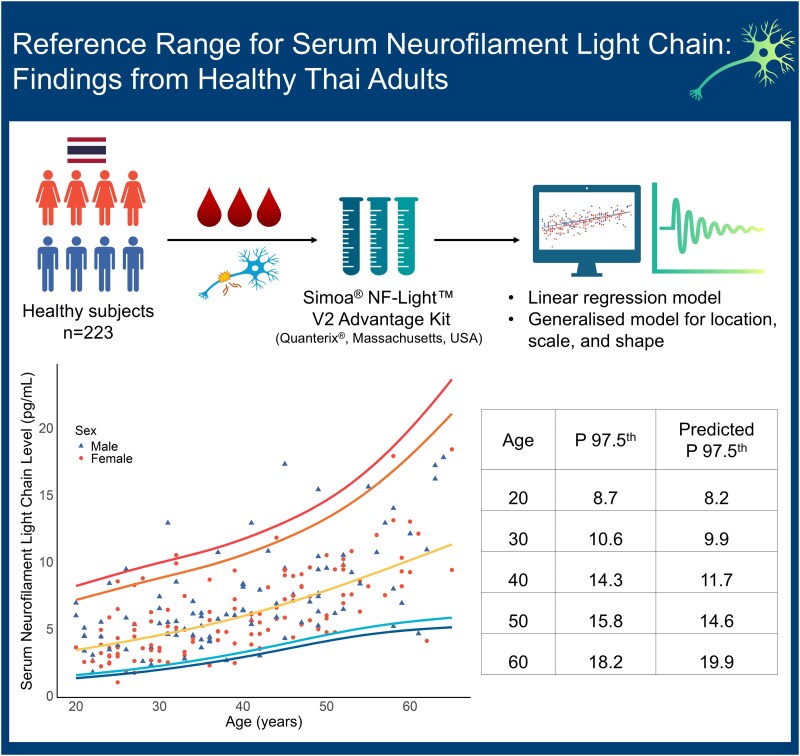

## Introduction

Over the past decades, accurately diagnosing and monitoring treatment responses in various neurological diseases has presented significant challenges. Thus, there is a compelling need for a reliable biomarker specific to neurological diseases that are easily tested. Neurofilament light chains (NfLs) have emerged as a biomarker of great interest among clinicians and researchers. NfL is a sub-unit of intermediate filament, which provides structural support for neurons and is particularly abundant in large, myelinated nerve fibres. An increase in NfL levels suggests axonal injury due to various causes, such as inflammation, neurodegenerative processes, traumatic brain injury and vascular insult, affecting both the CNS and peripheral nervous system. Beyond its diagnostic potential, studies have also highlighted the usefulness of NfL in predicting outcomes and assessing prognosis.^1,2^

Initially, NfL detection was mainly done in CSF.^3^ However, obtaining CSF samples is not always feasible. Recently, a method for measuring NfL in blood has been developed. An ultra-sensitive technique known as single-molecule array (Simoa®) now enables the detection of even very low levels of NfL that cross the blood–brain barrier into the bloodstream, pioneering the use of serum-based NfL as a biomarker. Serum NfL (sNfL) has shown potential for assisting the diagnosis and tracking the disease progression, for example, in multiple sclerosis,^4^ neurodegenerative diseases, such as Alzheimer’s disease^5^ and amyotrophic lateral sclerosis (ALS),^6^ epilepsy^7^ and traumatic brain injury.^8^ This serum-based NfL measurement paves the way for its use in clinical practice, including monitoring disease progression and treatment response and serving as an outcome measure in clinical trials. For example, it has been used as a marker of non-relapsing disease progression in multiple sclerosis in the ocrelizumab trial^9^ and as a marker of disease progression in *SOD1*-related ALS receiving the anti-sense oligonucleotide tofersen.^10^

While sNfL is sensitive to axonal loss in various neurological diseases, it lacks high specificity. Additionally, several patient factors such as age, body mass index (BMI) and other pre-existing health conditions could influence sNfL levels, such as chronic kidney disease.^11-13^ Age is considerably the strongest factor that increases the NfL concentration through age-related neurodegeneration mechanisms.^14^

Developing a reference value is the first milestone in implementing sNfL into clinical practice.^2^ A large reference database for sNfL levels in individuals without neurological disease has been published.^15^ However, sNfL concentrations are also varied across the populations.^16,17^ Hence, having a reference range for sNfL levels specific to the Thai population would be beneficial regarding the possibility of increasing the use of sNfL in clinical practice and expanding our current knowledge of sNfL reference values across populations. This pilot study aims to establish a reference interval and cut-off for sNfL in healthy individuals to provide a foundation for its use in clinical practice in Thailand.

## Materials and methods

### Participant and sample collection

Subjects were consecutively recruited from healthy blood donors at the Department of Transfusion Medicine, Siriraj Hospital, and healthy volunteers from April to July 2024. The inclusion criteria for participants were (i) age 18–70 years old, (ii) not having a history of neurologic diseases and a BMI <25 kg/m^2^ and (iii) being able to provide informed consent. Informed consent was given by all participants prior to the inclusion at Siriraj Hospital. Demographic details, including age and sex, were recorded. Patients were queried about their comorbidities, and those with the aforementioned conditions were excluded.

This study was approved by the Siriraj Institutional Review Board (certificate number SI064/2022).

### Laboratory handling of test samples

A 20-ml blood sample was collected from each participant at Siriraj Hospital, Bangkok, Thailand. Serum was extracted from the whole blood sample by centrifugation at 10 000 g for 5 min, immediately frozen at −20°C and subsequently stored at −80°C until the sNfL test was performed. All sera were tested for sNfL by clinically validated ultra-sensitive single-molecule array technology (Simoa® NF-Light™ V2 Advantage Kit, Quanterix®, Billerica, MA, USA). Prior to the analysis, the frozen serum samples were thawed at room temperature. The sera, along with duplicates, were then put into the 96-well plate, along with the calibrator and control provided with the kit for internal quality control. The plate and the reagent were put into the Simoa HD-X Analyser (Quanterix®) for the sNfL quantification,^18^ at the Neurology Laboratory, Division of Neurology, Department of Medicine, Siriraj Hospital, Thailand.

### Statistical analyses

The distribution of sNfL values was evaluated using a histogram, a Q–Q plot and the Shapiro–Wilk test. The sNfL levels were reported as medians with a 95% confidence interval (CI) computed using the bootstrap method. A comparison of medians between any two groups was done using the Mann–Whitney U-test. To examine the relationship between demographic variables and sNfL levels, a linear regression model was applied. The sNfL values, used as the outcome variable, were log_10_-transformed to ensure normality for the linear regression analysis.

The reference interval was defined as the range encompassing 95% of the values. The crude reference interval was determined based on the range between the 2.5th and 97.5th percentiles of sNfL values from all patients and within each 10-year age interval stratum, according to the Clinical and Laboratory Standards Institute (CLSI) guideline.^19^ To meet the minimum sample size requirement of 120 for reference range establishment, age groups of 20–39 (*n* = 115) and 40–69 (*n* = 108) were combined for analysis.

An age-normative reference curve for sNfL was developed using generalized additive model for location, scale and shape (GAMLSS) with the Box-Cox Cole and Green distribution family.^17^ The predicted percentile curves by age were generated and were further stratified by sex, including the 2.5th and 97.5th percentile curves, as pre-defined for reference interval by the CLSI guideline. In addition, a *Z*-score was also calculated for each data point using the formula for the Box-Cox Cole and Green distribution family:


$$z=\frac{{\left(\frac{y}{\mu }\right)}^{\nu }-1}{\nu \sigma }$$

Where *y* is the sNfL value, *µ* is the age-varying mean/median, *σ* is the coefficient of variance and *ν* is the skewness parameter.^20^

A *P*-value of <0.05 was considered statistically significant. All statistical analyses were conducted using R software, version 4.4.1.

## Results

### Value of serum neurofilament light chain in reference subjects

Blood samples were obtained from 223 Thai individuals, including 96 males (43.0%) and 127 females (57.0%), aged 18–70 years. The median age of participants was 39 years (interquartile range, 29–49). The sNfL levels showed a non-normal distribution. The median sNfL level was 5.8 pg/ml (95% CI 5.4–6.2), ranging from 1.0 to 18.4 pg/ml with a crude reference interval spanning the 2.5–97.5th percentiles of 2.3–15.9 pg/ml. Median sNfL levels were higher in males (*n* = 96; median = 6.1 pg/ml; 95% CI 5.5–6.9; reference interval: 2.6–16.8 pg/ml) compared with females (*n* = 127; median = 5.6 pg/ml; 95% CI 4.9–6.2; reference interval: 2.2–12.9 pg/ml), but the difference was not statistically significant (*P* = 0.056).

When stratified into 10-year age categories, a progressive increase in median levels was observed with advancing age. Patients aged 20–29 had the lowest median value of 3.6 pg/ml (95% CI 3.4–4.4), while those aged 60–69 exhibited the highest median at 12.1 pg/ml (95% CI 9.3–16.7). Similarly, the percentile ranges and the CIs of medians broadened with age, suggesting more pronounced variability in older groups. For instance, the 97.5th percentile increased from 8.7 pg/ml in the 20–29 age group to 18.2 pg/ml in the 60–69 age group. When grouped by the CLSI guidelines into broader categories, patients aged 20–39 years (*n* = 115) had a median of 4.4 pg/ml (95% CI 4.0–4.9), whereas those aged 40–69 years (*n* = 108) had a significantly higher median of 7.9 pg/ml (95% CI 7.2–8.5). This trend indicates an apparent age-related increase in levels, which may reflect physiological changes or other factors influencing the observed values. The medians and 2.5–97.5th percentile intervals of sNfL values in each age category are detailed in Table 1.

**Table 1:** Values and reference interval of sNfL in 223 healthy adults from Thailand

Age category	*n*	Median (95% CI) (pg/ml)	2.5th percentile (pg/ml)	97.5th percentile (pg/ml)
All patients				
20–65	223	5.8 (5.4–6.2)	2.3	15.9
10-year age category				
20–29	57	3.6 (3.4–4.4)	1.7	8.7
30–39	58	5.0 (4.5–5.6)	2.5	10.6
40–49	59	6.9 (6.1–7.6)	3.5	14.3
50–59	37	9.2 (7.6–9.8)	4.7	15.8
60–69	12	12.1 (9.3–16.7)	4.2	18.2
Age category with minimum requirement according to CLSI guideline				
20–39	115	4.4 (4.0–4.9)	1.76	9.97
40–69	108	7.9 (7.2–8.5)	3.73	17.5

### Prediction of the cut-off for serum neurofilament light chain

Linear regression analysis revealed that the male sex (*P* = 0.008) and older age (*P* < 0.001) were significantly associated with higher sNfL levels. The linear regression model for log sNfL was expressed as follows:


LogsNfL=(0.012×age)–(0.059×female)+0.326

**Figure 1:**
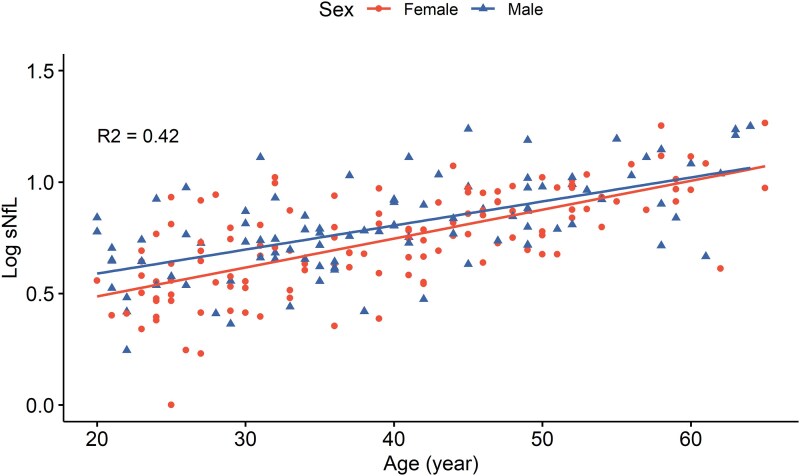
**Linear regression model of log-transformed sNfL and age, stratified by sex.** Linear regression model showing a significant correlation between sNfL and age (*n* = 223), controlling for sex (*P* < 0.001). The regression equation is Log sNfL = (0.012 × age) − (0.059 × female) + 0.326. Each data point represents an individual patient’s value, with colours indicating their sex.

Where 0.012 and −0.059 represent the coefficients for age and female sex, respectively, and 0.326 is the model intercept. Figure 1 demonstrates the correlation between log sNfL and age, with data stratified by sex.

While the standard error of the model was 0.166, the predicted 97.5th percentile of log sNfL was calculated as (0.012 × age)−(0.059 × female) + 0.326 + (1.96 × 0.166) at *Z* = 1.96. Therefore, the formula for the predicted 97.5th percentile of sNfL was obtained by exponentiating log sNfL, resulting in:


$$\mathrm{sNfL}=4.470\times 1.028^{\mathrm{age}}\;\times \;0.873^{\mathrm{female}}$$

**Figure 2:**
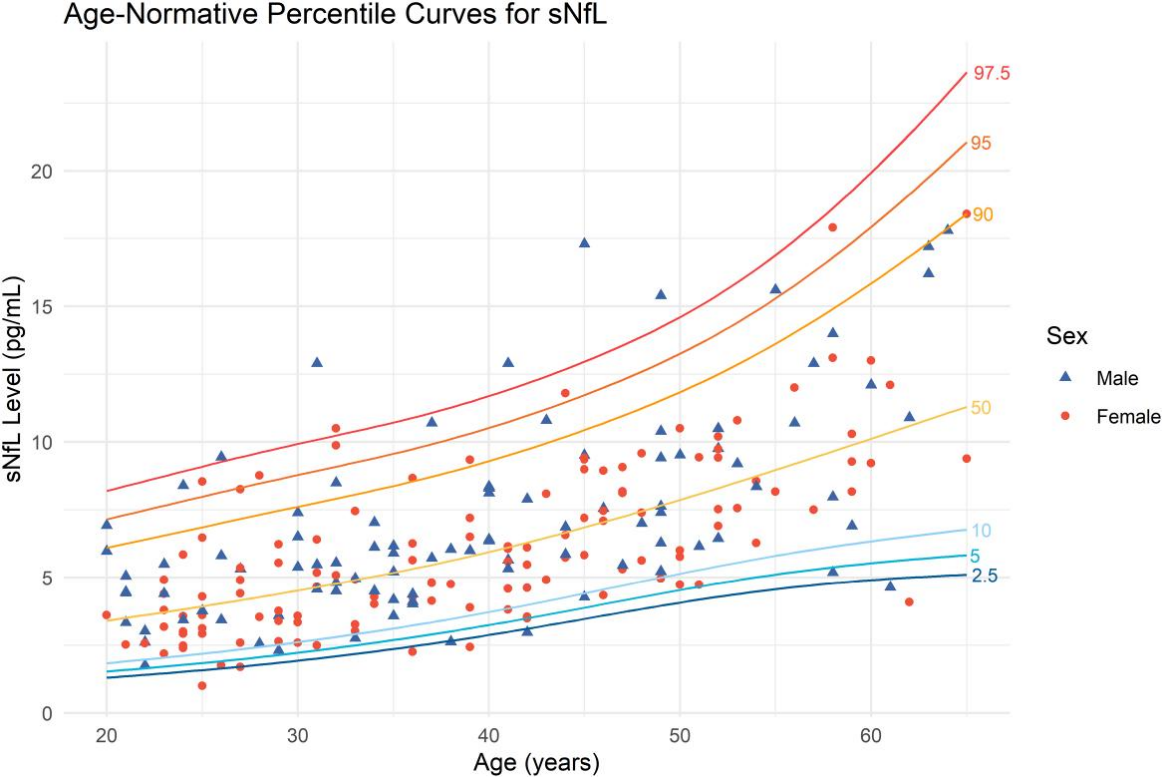
**GAMLSS-derived age-normative percentile curves for sNfL.** Age-normative percentile curves for sNfL derived from GAMLSS model applied on reference subjects (*n* = 223). Each data point represents an individual patient’s value, with colours indicating their sex. GAMLSS, generalized additive model for location, scale and shape.

**Figure 3:**
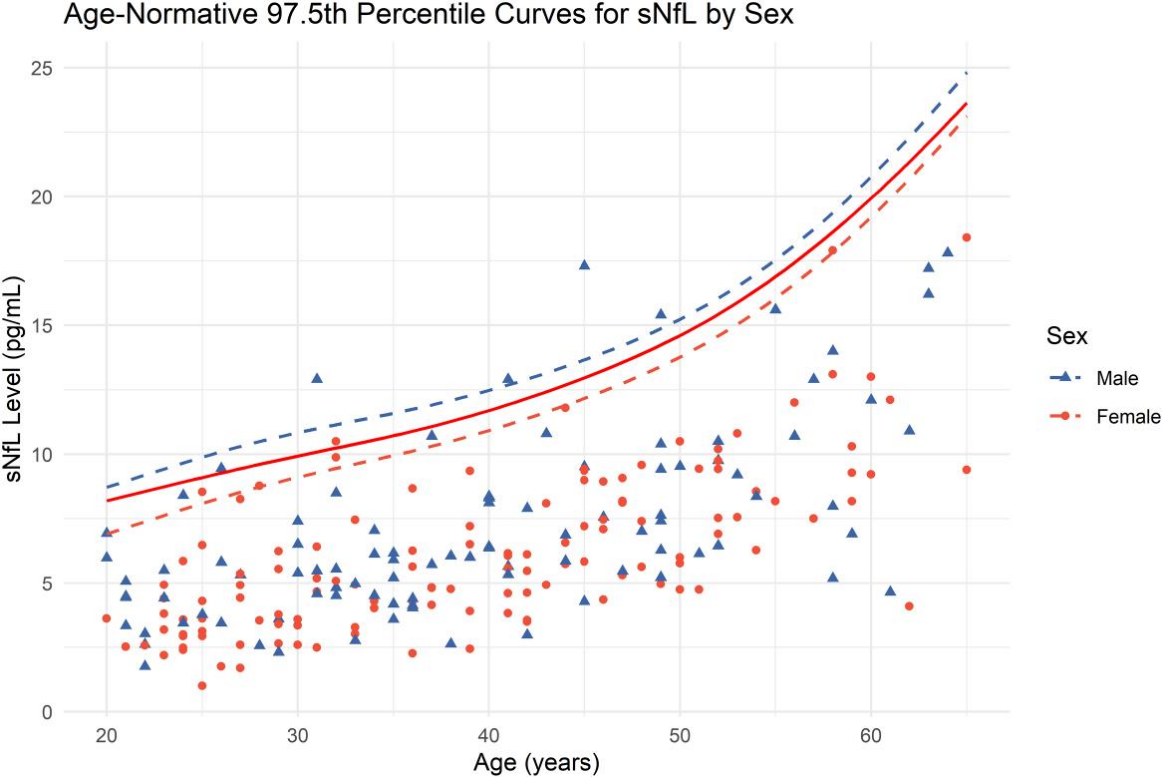
**GAMLSS-derived age-normative 97.5th percentile curve for sNfL, stratified by sex.** Age-normative 97.5th percentile of sNfL using GAMLSS model, stratified by sex (*n* = 223). Each data point represents an individual patient’s value, with colours indicating their sex.

The GAMLSS-derived age-specific percentile curves for sNfL and the 97.5th percentile curve for sNfL stratified by sex are presented in Figs 2 and 3. The predicted upper limit of sNfL values for various ages and sexes is summarized in Table 2, providing computed examples. Overall, the predicted sNfL levels closely matched the observed values, ranging from 8.2 to 19.9 pg/ml for ages 20–60. Predicted sNfL levels at the 2.5th, 5th, 10th, 50th, 90th, and 95th percentiles, stratified by age and sex, were also computed as a reference database (available in Supplementary Table 1). By visually inspecting the curve, the sNfL value increases exponentially after 50 years. A *Z*-score for the reference subject was computed using the results from the model and is presented in Supplementary Fig. 1. Overall, most of the data were distributed around the predicted median (*Z* = 0) for each age and sex, showing a normal distribution. Sixteen data points (7.2%) fall outside the interval between *Z* = −1.96 and 1.96, and nine data points (4.0%) are greater than *Z* = 1.96. When comparing the results from the linear regression model and GAMLSS, the difference in predicted sNfL levels was minimal, as presented in the Supplementary Table 2.

**Table 2:** Predicted 97.5th percentile of sNfL from the GAMLSS model

Age	Observed upper limit (pg/ml)	Predicted 97.5th percentile (pg/ml)		
		Total	Male	Female
20	8.68	8.2	8.7	6.9
30	10.6	9.9	10.8	9.1
40	14.3	11.7	12.5	10.9
50	15.8	14.6	15.2	13.8
60	18.2	19.9	20.8	19.2

### Comparison of serum neurofilament light chain values with previously published studies

Table 3 compares the characteristics and findings of selected studies measuring serum or plasma NfL levels across different age groups, of which two studies were from Asia: our study and Chen *et al*.^21^ All studies included subjects without documented neurological disorders and utilized the Simoa (Quanterix®) platform for NfL measurements.

**Table 3:** Summary of characteristics and reference values, by age, of NfL level from selected previous studies

Study	Number of subjects	Study site	Statistical analysis	Cut-off definition	NfL by age (pg/ml)				
					20	30	40	50	60
Chen *et al*.^21^	146	China	Subgroup descriptive analysis	P 95	8.4	9.2	22.2	34.1	68.3
Hviid *et al*.^22^	342	Denmark	Linear regression with log-transformed NfL	P 97.5	7.4	9.9	13.1	17.5	23.3
Bornhorst *et al*.^23^	1100	USA	Linear regression with log-transformed NfL	P 97.5	8.4	11.4	15.4	20.8	28.0
Benkert *et al*.^15^	5390	USA, Netherlands, Switzerland, Germany	GAMLSS and *Z*-score derivation	*Z* > 2.0 (P 97.72)^a^	8.5	11.5	14.2	18.1	23.8
Vermunt *et al*.^24^	833	Netherlands	Quantile regression with log-transformed NfL	P 95	9.0	10.0	12.0	14.0	19.0
Our study	223	Thailand	GAMLSS and *Z*-score derivation	P 97.5	8.2	9.9	11.7	14.6	20.8

P, percentile.

^a^The original study did not define a cut-off value; therefore, we selected *Z* > 2.0 to facilitate comparison with other studies using a similar definition. A BMI value of 25 kg/m^2^ was chosen as it is close to the average BMI of the Thai population.

Three studies used either quantile regression or linear regression on log-transformed NfL level,^22-24^ and two other studies used GAMLSS^15^ and descriptive analysis.^21^ Sample sizes varied from 146 to 5390 individuals. All studies had adjusted NfL values with age and showed a progressive increase in the NfL levels with increasing age.

Benkert *et al*.,^15^ with the largest sample size (*n* = 5390), derived *Z*-scores using GAMLSS and adjusted for age and BMI, defining the cut-off as *Z* > 2.0 (97.7th percentile). Their findings ranged from 8.5 pg/ml at 20 years to 23.8 pg/ml at 60 years. Similarly, Vermunt *et al*.^24^ used quantile regression to define the 95th percentile and reported slightly lower values, increasing from 9.0 pg/ml at age 20 to 19.0 pg/ml at age 60. Hviid *et al*.^22^ applied linear regression with log-transformed NfL, identifying the 97.5th percentile cut-off. Their results were comparable with those of Benkert *et al*.,^15^ with values ranging from 7.4 pg/ml at 20 years to 23.3 pg/ml at 60 years. Chen *et al*.,^21^ using descriptive subgroup analysis, demonstrated a dramatic increase in plasma NfL levels in older age groups, reaching 68.3 pg/ml at age 60. Bornhorst *et al*.^23^ focused on cognitively unimpaired individuals, also observed a consistent age-related increase, with 97.5th percentile levels rising from 8.4 pg/ml at 20 years to 28.0 pg/ml at 60 years. A full detailed table describing study characteristics can be found in Supplementary Table 3.

## Discussion

In this study, we demonstrated a significant association between sNfL levels and both age and sex. Leveraging data from 223 healthy Thai adults, we proposed a reference limit for sNfL stratified by age and sex for the Thai population using 2 complementary methods: the conventional descriptive 2.5th and 97.5th percentiles for each 10-year age group and a GAMLSS-derived age-normative reference curve.

Our findings revealed that age and male sex were significantly correlated with increasing sNfL levels, with age exhibiting a stronger association. Consistent with previous studies, age emerged as a dominant factor influencing sNfL elevation, likely due to age-related neurodegenerative processes.^14^ Uniquely, we also observed a significant impact of sex, wherein females demonstrated a median sNfL level of ∼1 pg/ml lower than males. While most prior studies proposing NfL reference intervals did not stratify by sex, a few have reported similar sex-related differences, albeit with smaller effect sizes.^18,25^ Given these results, we computed an age-normative reference curve stratified by sex.

Other factors, such as BMI, glycated haemoglobin, serum creatinine and liver function markers, have been shown to correlate significantly with sNfL levels.^15,25^ These variables were not included in our model as potential confounders. However, all subjects in our cohort had BMIs below 25 kg/m^2^, consistent with the Thai population average,^26^ and were free of underlying diseases at inclusion.

Using the GAMLSS model, we derived an age-normative reference curve for sNfL. GAMLSS is a powerful statistical tool increasingly utilized in biomarker research, as it enables modelling beyond the mean by accounting for variance, skewness and kurtosis. Its flexibility allows for the robust handling of non-normally distributed variables and outliers while capturing non-linear relationships.^27^ Notably, GAMLSS has been widely adopted in clinical practice, with applications such as growth curve modelling.^20^ This method is particularly suitable for sNfL, a variable often skewed and age dependent, and addresses challenges posed by small sample sizes when stratifying subjects in a 10-year age interval.

In our cohort of healthy Thai adults, the 97.5th percentile of sNfL ranged from 6.9 to 20.8 pg/ml, progressively increasing with age. Our results are slightly lower but not considerably different compared with previous studies reporting an upper limit of ∼10 pg/ml in individuals around 20 years old, rising to more than 20 pg/ml by age 60, summarized in Table 3.^15,18,21-24^ A more pronounced difference is observed above 50 years. Similar to earlier studies, a visual inspection of our curve revealed a steeper increase in sNfL levels after the age of 50.^15^ This consistency across studies highlights the critical importance of accounting for age when interpreting sNfL levels. However, it is worth noting that our study has a limited sample size of subjects above 50 years old, making the estimation less accurate.

The results from the GAMLSS model also allowed for the computation of *Z*-scores, providing a standardized method to quantify deviations from the central values, which is more clinically interpretable than the discrete percentile-based cut-offs. Since the reference cohort itself was used to compute the *Z*-scores, they were mainly distributed around *Z* = 0 (Supplementary Fig. 1). This behaviour aligns with the theoretical expectation that the observed values should closely match the predicted mean or median for ages for a well-fitted model. This finding confirmed that the model was reasonably well fitted, and *Z*-scores could be derived from the predicted medians, standard deviations and kurtosis for different ages. Nonetheless, external validation in patients with neurological diseases remains essential to ensure real-world applicability.

Our study is the first to establish normative sNfL values in a healthy Thai population, paving the way for its potential clinical application in Thailand. We employed GAMLSS, a robust statistical method capable of accommodating the skewed distribution of sNfL while accounting for outliers, variance and non-linear relationships. However, we acknowledge certain limitations. Our sample size, though adequate, is smaller than in other studies, particularly for individuals older than 50, potentially reducing the precision of estimates in this age group. Furthermore, while our cohort comprised subjects with normal BMI and no pertinent underlying diseases, we did not adjust for potential confounders such as serum creatinine, glycated haemoglobin and BMI, which are known to influence sNfL levels. Last, our reference model has not yet been validated in patients with neurological disease, though a validation study is planned.

In summary, this study is the first to explore sNfL levels in healthy Thai adults and establish age- and sex-specific reference intervals. In addition to age, we identified a significant association between sex and sNfL levels, with females exhibiting lower sNfL concentrations. The GAMLSS-derived 97.5th percentile curve provided a robust and clinically relevant reference model, yielding values consistent with prior studies. This pilot work lays the foundation for future clinical applications and biomarker research involving sNfL in Thailand. In the next steps, we plan to test these reference values in real clinical settings, first in patients with CNS demyelinating diseases, including multiple sclerosis and neuromyelitis optica spectrum disorders.

## Supplementary Material

fcaf166_Supplementary_Data

## Data Availability

Anonymized raw data are available from the corresponding author upon reasonable request. The code generated for this work can be found at https://github.com/nontapatsuk/Thai-NfL-ref.git.
