# Supplementary material for: Reference range for serum neurofilament light chain: findings from healthy Thai adults
Source: Brain Commun. 2025 Apr 29;7(3):fcaf166. doi: 10.1093/braincomms/fcaf166 (PMC12062521; doi:10.1093/braincomms/fcaf166)
Supplement: fcaf166_Supplementary_Data [file fcaf166_supplementary_data.pdf]

Supplementary Materials

Supplementary Table 1 Predicted sNfL concentration corresponding to a certain percentile at a given age and sex from the GAMLSS model applied to the Thai reference subjects

Age	Sex	sNfL level at a certain percentile; pg/mL						
		**2.5th**	**5th**	**10th**	**50th**	**90th**	**95th**	**97.5th**
20	Male	1.56	1.82	2.16	3.85	6.63	7.69	8.72
	female	1.24	1.44	1.71	3.05	5.25	6.09	6.91
25	Male	1.82	2.11	2.50	4.42	7.54	8.73	9.88
	female	1.49	1.73	2.05	3.62	6.18	7.14	8.09
30	Male	2.16	2.48	2.91	5.02	8.36	9.62	10.8
	female	1.81	2.09	2.45	4.22	7.03	8.08	9.11
35	Male	2.60	2.96	3.43	5.66	9.10	10.4	11.6
	female	2.23	2.54	2.94	4.86	7.82	8.90	9.95
40	Male	3.13	3.53	4.05	6.43	9.97	11.3	12.5
	female	2.74	3.09	3.54	5.63	8.73	9.85	10.9
45	Male	3.76	4.20	4.76	7.33	11.1	12.4	13.7
	female	3.35	3.74	4.24	6.53	9.86	11.0	12.2
50	Male	4.37	4.86	5.50	8.34	12.4	13.9	15.2
	female	3.95	4.40	4.97	7.54	11.2	12.5	13.8
55	Male	4.84	5.41	6.13	9.43	14.2	15.9	17.5
	female	4.43	4.95	5.61	8.63	13.0	14.6	16.1
60	Male	5.08	5.74	6.60	10.6	16.5	18.7	20.8
	female	4.70	5.31	6.10	9.77	15.3	17.3	19.2
65	Male	5.16	5.92	6.92	11.7	19.3	22.1	24.8
	female	4.81	5.52	6.45	10.9	18.0	20.6	23.1

Abbreviations: sNfL, serum neurofilament light chain, GAMLSS, generalized additive model for location, scale, and shape

Supplementary Table 2 Comparison of predicted sNfL levels from the regression model and GAMLSS.

Age	Predicted sNfL by regression model (pg/mL)*			Predicted sNfL by GAMLSS (pg/mL)		
	Total	Male	Female	Total	Male	Female
20	7.2	7.8	6.8	8.2	8.7	6.9
30	9.6	10.2	8.9	9.9	10.8	9.1
40	12.6	13.5	11.8	11.7	12.5	10.9
50	16.6	17.8	15.5	14.6	15.2	13.8
60	22.0	23.5	20.4	19.9	20.8	19.2

Abbreviation: sNfL, serum neurofilament light chain; GAMLSS, generalized additive model for location, scale, and shape

*The predicted sNfL is calculated from an exponential linear regression equation of Log NfL at 97.5th percentile: Log NfL = (0.012 × Age) – (0.059 × Female) + 0.326 + (1.96 × 0.166), whereas 0.166 is a standard error of the model and Z=1.96, which is equal to: sNfL = $4.470 \times 1.028^{Age} \times 0.873^{Female}$.

Supplementary Table 3 Detail summary of characteristics and reference values, by age, of NfL level from selected previous studies

Study	Participant characteristics	Number of subjects	Study site	Measurement method	Statistical analysis	Adjustment variable	Cut-off definition	NfL by age (pg/mL)				
								20	30	40	50	60
Chen, et al. (2021)[1]	Subjects without underlying disease or a neurological disorder	146	China	Plasma, Simoa (Quanterix®)	Subgroup descriptive analysis	Age	P 95	8.4	9.2	22.2	34.1	68.3
Hviid, et al. (2020)[2]	Blood donors	342	Denmark	Serum, Simoa (Quanterix®)	Linear regression with log-transformed NfL	Age	P 97.5	7.4	9.9	13.1	17.5	23.3
Bornhorst, et al. (2022)[3]	Cognitive-unimpaired subjects without known chronic kidney disease, stroke or myocardial infarction, and BMI <30	1100	USA	Plasma, Simoa (Quanterix®)	Linear regression with log-transformed NfL	Age	P 97.5	8.4	11.4	15.4	20.8	28.0
Benkert, et al. (2022)[4]	Subjects without a neurological disorder	5,390	USA Netherlands Switzerland Germany	Serum, Simoa (Quanterix®)	GAMLSS and Z score derivation	Age, BMI	Z > 2.0 (P 97.72)*	8.5	11.5	14.2	18.1	23.8
Vermunt, et al. (2022)[5]	Subjects without a neurological disorder	833	Netherlands	Serum, Simoa (Quanterix®)	Quantile regression with log-transformed NfL	Age	P 95	9.0	10.0	12.0	14.0	19.0
Our study	Subjects without a neurological disorder	223	Thailand	Serum, Simoa (Quanterix®)	GAMLSS and Z score derivation	Age, sex	P 97.5	8.2	9.9	11.7	14.6	20.8

Abbreviation: BMI, body mass index; GAMLSS, generalized additive model for location, size, and shape; P, percentile

* The original study did not define a cut-off value; therefore, we selected Z > 2.0 to facilitate comparison with other studies using a similar definition. A BMI value of 25 kg/m² was chosen, as it is close to the average BMI of the Thai population.

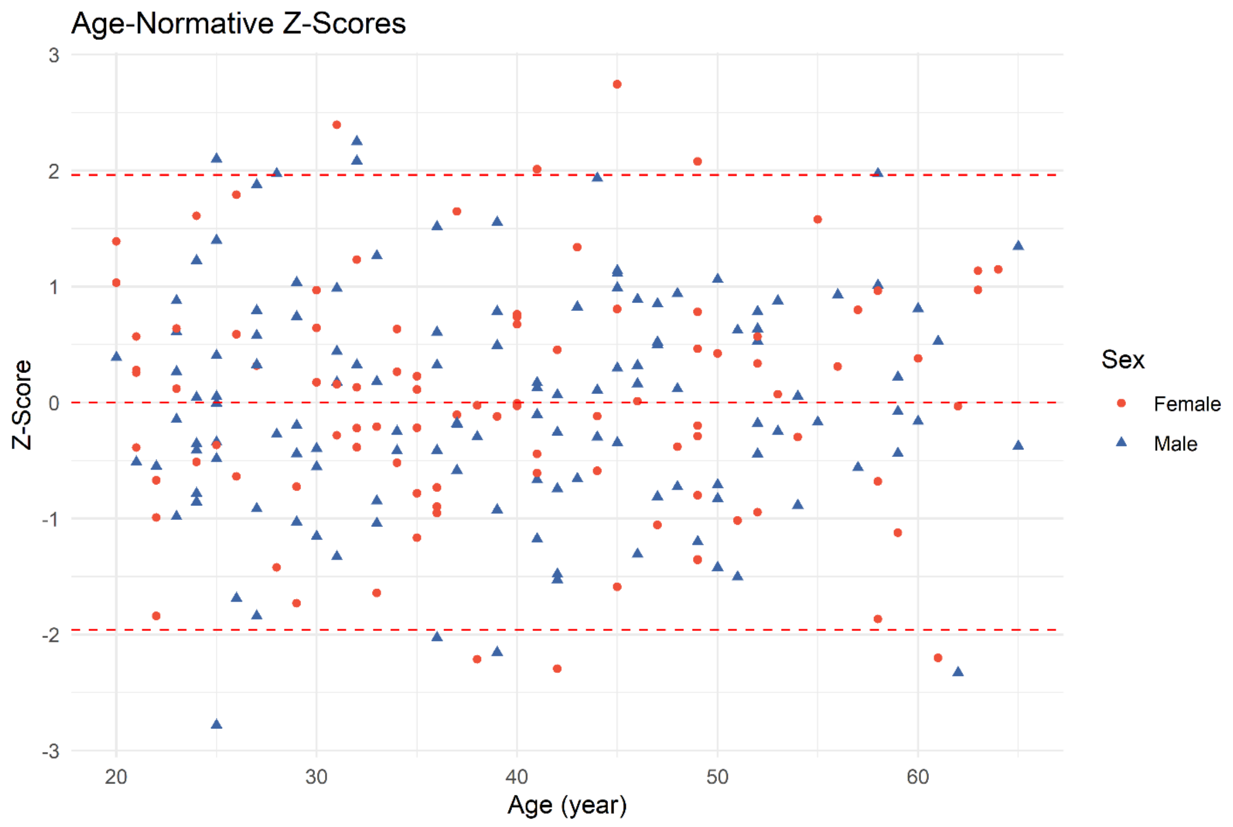

Supplementary Figure 1 Z-score calculation of sNfL from the reference subjects.

Distribution of age-normative Z-scores derived from the output of GAMLSS, stratified by sex (n=223). Each point represents a Z-score derived from each sNfL value.

Abbreviations: GAMLSS, generalized additive model for location, scale, and shape; sNfL, serum neurofilament light chain

Reference

1. Chen J, Yang X, Zhang Y*, et al*. Reference values for plasma neurofilament light chain (NfL) in healthy Chinese. *Clin Chem Lab Med*. Mar 26 2021;59(4):e153-e156. doi:10.1515/cclm-2020-1030
2. Hviid CVB, Knudsen CS, Parkner T. Reference interval and preanalytical properties of serum neurofilament light chain in Scandinavian adults. *Scand J Clin Lab Invest*. Jul 2020;80(4):291-295. doi:10.1080/00365513.2020.1730434
3. Bornhorst JA, Figdore D, Campbell MR*, et al*. Plasma neurofilament light chain (NfL) reference interval determination in an Age-stratified cognitively unimpaired cohort. *Clin Chim Acta*. Oct 1 2022;535:153-156. doi:10.1016/j.cca.2022.08.017
4. Benkert P, Meier S, Schaedelin S*, et al*. Serum neurofilament light chain for individual prognostication of disease activity in people with multiple sclerosis: a retrospective modelling and validation study. *Lancet Neurol*. Mar 2022;21(3):246-257. doi:10.1016/s1474-4422(22)00009-6
5. Vermunt L, Otte M, Verberk IMW*, et al*. Age- and disease-specific reference values for neurofilament light presented in an online interactive support interface. *Ann Clin Transl Neurol*. Nov 2022;9(11):1832-1837. doi:10.1002/acn3.51676
